# Systematic Analysis of the Multiple Bioactivities of Green Tea through a Network Pharmacology Approach

**DOI:** 10.1155/2014/512081

**Published:** 2014-11-30

**Authors:** Shoude Zhang, Lei Shan, Qiao Li, Xia Wang, Shiliang Li, Yuan Zhang, Jianjun Fu, Xiaofeng Liu, Honglin Li, Weidong Zhang

**Affiliations:** ^1^Shanghai Key Laboratory of New Drug Design, State Key Laboratory of Bioreactor Engineering, School of Pharmacy, East China University of Science and Technology, Shanghai 200237, China; ^2^School of Pharmacy, Second Military Medical University, Shanghai 200433, China

## Abstract

During the past decades, a number of studies have demonstrated multiple beneficial health effects of green tea. Polyphenolics are the most biologically active components of green tea. Many targets can be targeted or affected by polyphenolics. In this study, we excavated all of the targets of green tea polyphenolics (GTPs) though literature mining and target calculation and analyzed the multiple pharmacology actions of green tea comprehensively through a network pharmacology approach. In the end, a total of 200 *Homo sapiens* targets were identified for fifteen GTPs. These targets were classified into six groups according to their related disease, which included cancer, diabetes, neurodegenerative disease, cardiovascular disease, muscular disease, and inflammation. Moreover, these targets mapped into 143 KEGG pathways, 26 of which were more enriched, as determined though pathway enrichment analysis and target-pathway network analysis. Among the identified pathways, 20 pathways were selected for analyzing the mechanisms of green tea in these diseases. Overall, this study systematically illustrated the mechanisms of the pleiotropic activity of green tea by analyzing the corresponding “drug-target-pathway-disease” interaction network.

## 1. Introduction

**Figure 1:**
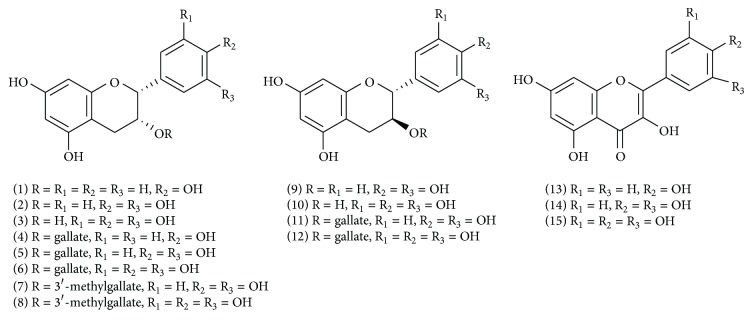
Structures of green tea polyphenols.

Tea is a traditional medicinal plant and the most widely consumed beverage in the world. Among all teas consumed worldwide, green tea is the best studied in terms of health benefits because its chemistry is more well known than that of other teas [1]. A number of studies have demonstrated the beneficial health effects of green tea, which include the reduction of serum cholesterol, the prevention of low-density lipoprotein oxidation, and a decreased risk of cardiovascular disease and cancer [2, 3]. It is generally agreed that many of the effects of green tea are mediated by its polyphenols, as shown in Figure 1, which include flavanols and flavonoids. The flavanols, which are also known as catechins, including (−)-epiafzelechin (EZ,** 1**), (+)-catechin (C,** 9**), (−)-epicatechin (EC,** 2**), (+)-gallocatechin (GC,** 10**), (−)-epigallocatechin (EGC,** 3**), their respective 3-gallate esters (−)-EZG (**4**), (+)-CG (**11**), (−)-ECG (**5**), (+)-GCG (**12**), and (−)-EGCG (**6**), and two 3-(3′-methy)gallate esters (−)-ECMG (**7**) and (−)-EGCMG (**8**), account for 40–50% of the dry weight of tea leaves [4, 5]. In addition, three flavonoids, namely kaempferol (**13**), quercetin (**14**), and myricetin (**15**), have been isolated as components of green tea [6]. The diverse bioactivities of green tea have prompted comprehensive research, which has led to hundreds of published studies. However, most of these studies mainly focused on the anticancer activity of EGCG, a major catechin of green tea. Such one-sided studies are not aligned with the diverse bioactivities of green tea. In addition, many of the other GTPs also have been proven to present multiple bioactivities, such as the kaempferol and quercetin, which exert potentially beneficial effects on inflammation [7]. Moreover, GTPs often play target multiple proteins. For example, EGCG has been shown to mediate multiple signal pathways by binding to many targets in cancer cells [8]. Therefore, it is reasonable to systematically and comprehensively analyze the mechanisms of action of green tea based on the “multicomponent, network target” model.

Network pharmacology updates the research paradigm from the current “one target, one drug” model to a new “multicomponent, network target” model, which enhances our knowledge of multipathway interactions and helps interpret drug-response signature datasets that collectively decode the complex mechanisms of drug actions [9]. Unlike earlier reductionist “one drug, one target” approaches, network pharmacology invokes the idea that a drug engages with multiple targets and rarely interacts with a single protein in isolation [10]. This approach utilizes principles of systems biology and network analysis to advance drug discovery through the identification of connectivity, redundancy, and pleiotropy in biological pathways and has allowed a deeper understanding of the drug interactions by revealing that this promiscuity often engages a synergistic combination of appropriate high-value targets in a complex disease, such as cancer and diabetes, to produce treatment success [11].

**Figure 2:**
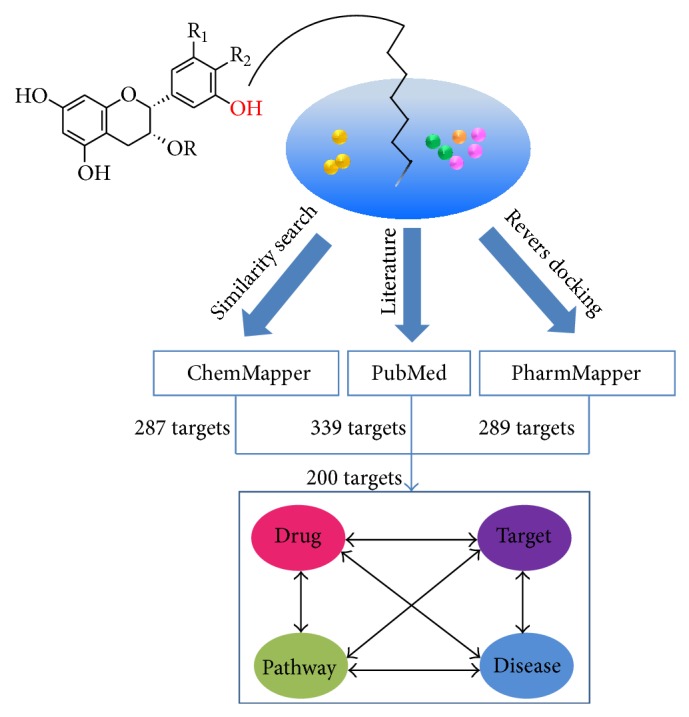
Analysis overview. The methods of similarity search, literature mining, and reverse docking were utilized to identify the confirmed and potential targets of GTPs, and these methods provides 287, 339, and 289 targets, respectively. Ultimately, 200* Homo targets* were selected after removing any redundant and other* sapiens* targets and used to construct the “drug-target-pathway-disease” interaction network.

To explore the mechanism of action of green tea from a holistic perspective, the network pharmacology method was employed to investigate the molecular behavior of GTPs. We collected all available target information for fifteen GTPs through literature mining and computational chemistry (3D similarity search and reverse docking) to construct a network of the “drug-target-pathway-disease” interactions (Figure 2). In the end, 200* Homo sapiens* targets were identified for fifteen GTPs. These targets were classified into six groups according to their related disease, which included cancer, diabetes, neurodegenerative disease, cardiovascular disease, muscular disease, and inflammation, in agreement with the applications of green tea. Pathways analysis was used to illustrate the mechanism of action through which the GTPs exert their comprehensive therapeutic effects. A total of 143 KEGG pathways were identified, and 26 of these were more enriched, as determined through pathway enrichment analysis and target-pathway network analysis. Twenty of the identified pathways that play a role in the GTP-related diseases were selected for analyzing the mechanisms of action of green tea. By integrating the “drug-target-pathway-disease” interactions, we found the green tea exerts a variety of therapeutic effects though the modulation of multiple pathway by its main components, which is in accordance with the “multicomponent, network target” model.

## 2. Material and Methods

### 2.1. Literature Mining

All available information on the targets of fifteen GTPs in the literature was collected by searching PubMed using the Structure search. Only the confirmed and active targets were selected from the category of biological test results.

### 2.2. Similarity Search

The online web server ChemMapper [12], which is based on the 3D similarity procedure ShAFTS, was used to predict the potent targets according to the similar property principle, which suggests that structurally similar molecules should exhibit the same (or similar) bioactivities [13]. SHAFTS provides a ShapeScore (based on the shape overlap) and a FeatureScore (based on the pharmacophore fit), and the weighted sum of the two scores is considered the hybrid similarity HybridScore [14]. A higher HybridScore implies a better alignment in terms of both shape and chemotype identities between the query and target molecules [15]. In this study, the targets with a HybridScore value higher than 1.400 were selected as potent targets.

### 2.3. Reverse Docking

Reverse docking is a novel technology that allows the docking of a compound with a known biological activity into the binding sites of all of the 3D structures in a given protein database. The PharmMapper server is a freely accessed web server designed to identify potential target candidates for a specific small molecule probe (drugs, natural products, or other newly discovered compounds with unidentified binding targets) using the pharmacophore mapping approach [16]. It is backed up by a database with a large repertoire of pharmacophores extracted from all of the targets in TargetBank, DrugBank, BindingDB, and PDTD. More than 7,000 receptor-based pharmacophore models (covering 1,627 drug targets, 459 of which are human protein targets) are stored and accessed by PharmMapper. This program finds the best mapping poses of the user-uploaded molecules against all of the targets in PharmTargetDB, and the top N potential drug targets, as well as the respective molecules' aligned poses, are outputted. In this study, the cutoff Fit Score value was set to 4.000. The targets with a Fit Score value higher than 4.000 were selected as potential targets.

### 2.4. Network Construction and Analysis

The individual interaction networks for each protein were built by use of the STRING database which is integration of known and predicted protein interactions [17]. The network interactions were selected according to STRING-computed confidence scores (medium confidence 0.4000). The Cytoscape software (Version 2.8.3; http://www.cytoscape.org/) and the Network Analyzer plugin (Version 1.0, http://med.bioinf.mpi-inf.mpg.de/netanalyzer/) were used to visualize the network and calculate the basic network parameters, including degree of distribution, degree exponent, shortest-path-length distribution, and clustering coefficient. The interactions in the “target-pathway” network were selected from the pathway enrichment results. The sizes of the nodes correspond to the node degree.

### 2.5. Pathway Enrichment

*P* values were used to determine if a specific pathway in the KEGG database was more enriched with the related proteins than by chance. Assuming that a total of *K* proteins related to the GTPs were mapped into KEGG, which contains *N* distinct proteins, and *k* proteins from a pathway of size *n* are related to the GTPs, the *P* value is given by

(1) $$\begin{array}{c} P=1-\overset{k-1}{\underset{i=0}{\sum }}f\left(i\right)=1-\overset{k-1}{\underset{i=0}{\sum }}\frac{\left(\begin{array}{c} K\\ i \end{array}\right)\left(\begin{array}{c} N-K\\ n-i \end{array}\right)}{\left(\begin{array}{c} N\\ n \end{array}\right)}. \end{array}$$

The two-sided hypergeometric test and the Bonferroni correction were used.

## 3. Results and Discussion

### 3.1. Data Preparation

The 15 GTPs shown in Figure 1 were searched by PubMed, ChemMapper, and PharmMapper. From the results from the PubMed search, only the active and confirmed targets were selected, resulting in the identification of 339 targets for the 15 components. The web service ChemMapper, which is based on a 3D similarity search, provided 287 targets with a HybridScore value of at least 1.400, and the web service PharmMapper provided 289 targets with a Fit Score value of at least 4.000. Because polyphenols have a similar skeleton, most of these compounds often share the same targets. After removing any redundant and other* sapiens* targets, 200* Homo sapiens* targets were selected for the subsequent analysis (see Table S1 in the Supplementary Material available online at http://dx.doi.org/10.1155/2014/512081).

### 3.2. Drug-Target Interactions

**Figure 3:**
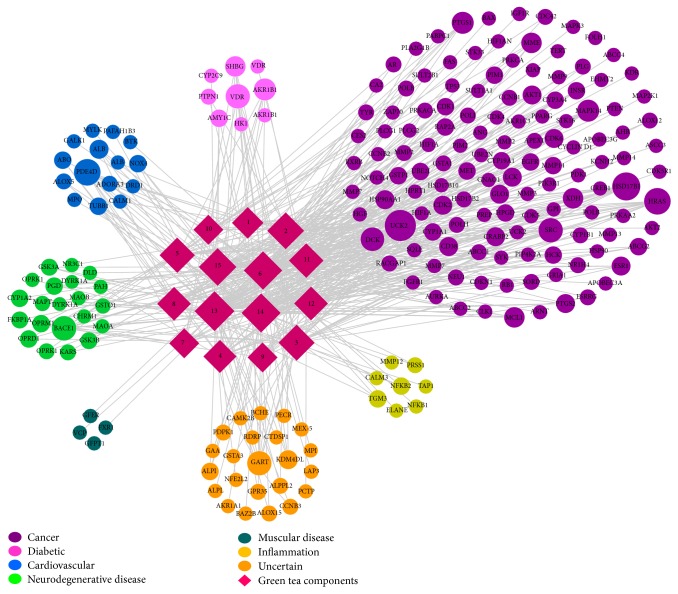
The drug-target-disease network. Two-hundred* Homo sapiens* targets targeted by fifteen GTPs were classified into seven groups according to their related disease, as determined by the Disease and Gene Annotations. The circles represent the targets, and the rhombi represent the GTPs.

The network of drug-target interactions is shown in Figure 3. The target proteins were classified into six groups according to their related disease, as determined using the database of Disease and Gene Annotations (DGA) [18]. Among all 200* Homo sapiens* targets, 120 targets were related to cancer, which is in agreement with previous research results showing that green tea has anticancer activity [19]. In addition, the other 80 targets were associated with diabetes, cardiovascular disease, Alzheimer's disease, muscular disease, mental disease, and inflammation. The sizes of the nodes correspond to the degree. It was easily determined that most of the GTPs shared multiple targets, particularly** 13** (kaempferol),** 14** (quercetin),** 6** ((−)-EGCG), and** 15** (quercetin), which exhibited node degrees greater than 40. Simultaneously, most of the targets can be targeted by more than one GTP.

### 3.3. Protein-Protein Interactions

**Figure 4:**
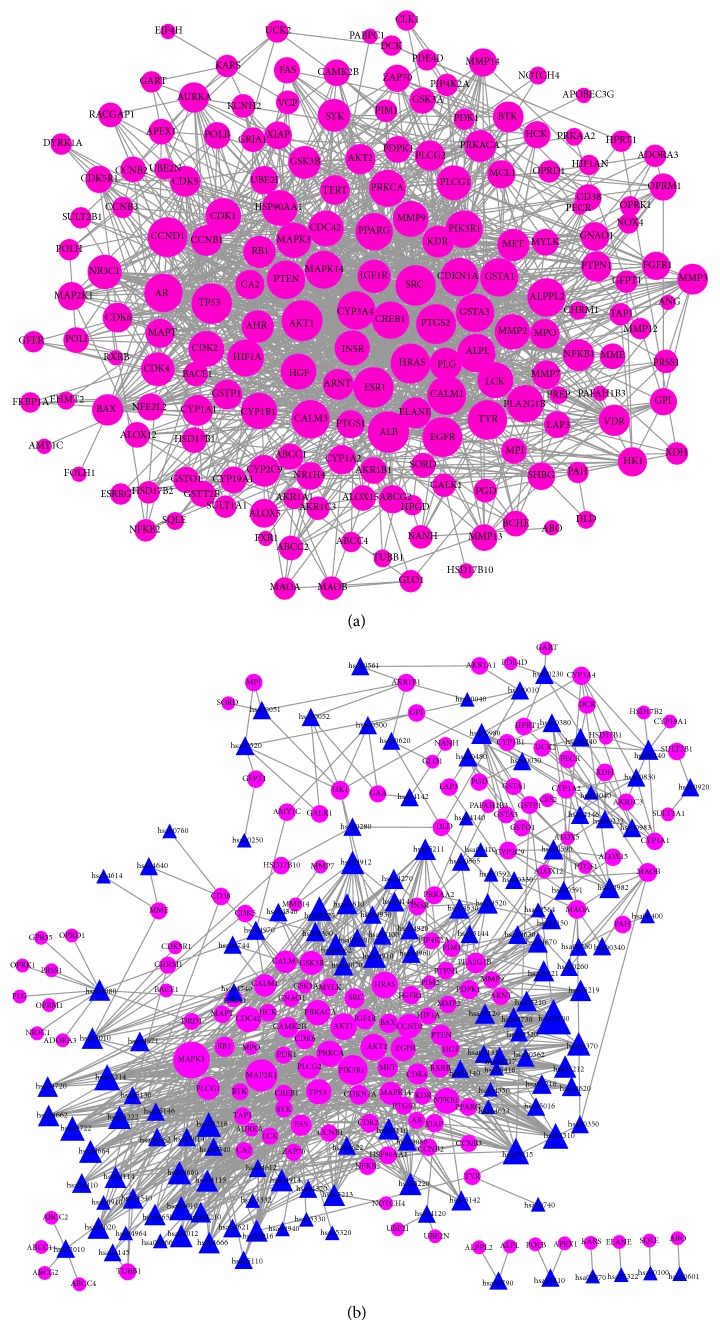
Protein-protein network and protein-pathway network. (a) Protein-protein network. The proteins are colored pink and shown as circles. (b) Protein-pathway network. The proteins are colored pink and shown as circles, and the pathways are colored blue and shown as triangles. All of the graphs are shown through an organic layout, and the node size corresponds to the degree.

The protein-protein network was constructed* via* mapping the 200 putative targets into the String database, which is a collection of known and predicted protein-protein interactions. After excluding isolated nodes, the protein interaction network induced by green tea components was composed of 187 nodes (proteins) and 1500 edges (interactions) (Figure 4(a), Table S1). The topological properties of the network rewired by the GTPs were analyzed with the network analyzer plugin. Among these properties, the node degree can be used to distinguish between random and scale-free network topologies. In a random network, the node degrees often follow a Poisson distribution, whereas these exhibit a nonuniform distribution in a scale-free network. Most network models based on a biological system are scale-free. In the network rewired by GTPs, the node degree distribution was in accordance with a power law, indicating that the constructed network is scale-free and does not present a random topology (Figure S1).

### 3.4. Pathway Enrichment

Because green tea exhibits diverse bioactivities mediated by multiple pathways, it is reasonable to analyze the potential pathways. In this study, pathway enrichment based on the hypergeometric test was utilized to analyze the potential pathways mediated by the GTPs. Ultimately, 143 KEGG pathways were identified, and 26 of these were found to be more enriched (*P* value < 0.05, Table 1, Table S2). These results were confirmed by constructing the protein-pathway network. As shown in Figure 4(b), the pathways with a lower *P* value exhibited a larger node size, that is, a greater node degree; thus, the five pathways with the greatest degrees, namely, hsa05200, hsa05215, hsa05214, hsa04510, and hsa05218, also presented the six highest *P* values. In addition, because green tea has previously been associated with cancer, diabetes, cardiovascular diseases, neurodegenerative disease, muscular disease, and inflammation, 20 pathways related with these diseases were selected for further analysis (Table 2).

### 3.5. Anticancer Mechanism

Anticancer is one of most commonly reported effect of green tea. Numerous studies have provided evidence that green tea has potential chemotherapeutic activity against a wide range of cancers, including those involving the skin, lungs, gastrointestinal tract, breast, colon, and the head and neck [20]. Extensive research studies have attempted to elucidate the molecular mechanisms of cancer chemoprevention by green tea. However, these studies have mainly focused on the anticancer activity of EGCG, and its targeting of specific cell signaling pathways has received considerable attention for the regulation of cellular proliferation and apoptosis. Recent studies have shown that EGCG can target multiple signaling pathways in cancer, including the epidermal growth factor receptor (EGFR), insulin-like growth factor (IGF), mitogen-activated protein kinase (MAPK)/extracellular signal-regulated kinase (ERK), and NF-*κ*B pathways [8, 21]. Although EGCG is the main polyphenol and has higher anticancer activity, the other GTPs, such as kaempferol [22] and quercetin [23], also present anticancer activity. Therefore, a systematic analysis of the anticancer mechanism of all GTPs is necessary.

**Figure 5:**
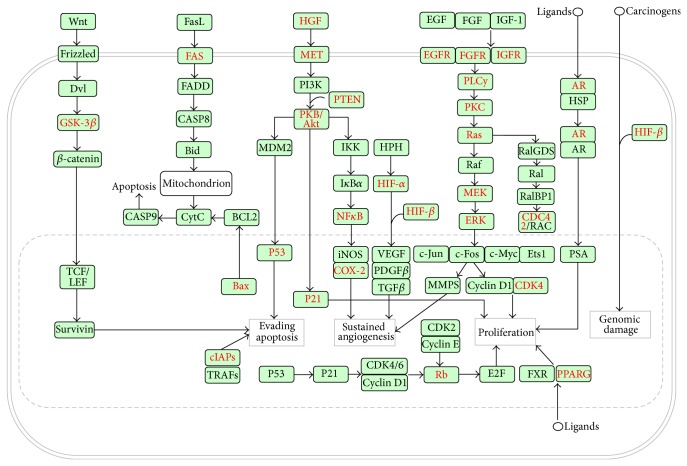
Simplified pathways in cancer. All of the targets are shown by the gene name. The GTP-regulated targets are labeled in red.

To fully elucidate the molecular mechanisms in cancer prevention, we collected all of the potential and confirmed targets of the GTPs to identify all of the related pathways. In the end, 12 pathways in cancer were identified through pathway enrichment analysis (Table 2). To better understand the anticancer mechanisms of green tea, we constructed a simplified pathway covering most of the targets related to the GTPs based on the hsa05200 pathway (pathways in cancer), which is an integration of other pathways, including glioma, prostate cancer, non-small-cell lung cancer, melanoma, pancreatic cancer, endometrial cancer, bladder cancer, small cell lung cancer, acute myeloid leukemia, colorectal cancer, and renal cell carcinoma (Figure 5). Our analysis revealed a total of 29 targets that are modulated by the polyphenols of green tea (colored in red). These were distributed in different signaling pathways and regulated by one or more polyphenols (Table S2). GSK-3*β* belongs to the Wnt signaling pathway and was modulated by compounds** 13**,** 14**, and** 15**. FAS and Bax belong to the apoptosis signaling pathway and were modulated by compounds** 5** and** 6**, respectively. These pathways affect the growth of cancer cells by regulating their apoptosis. In addition, the targets cIAPs and P53 were also related with apoptosis. The other targets modulated by GTPs were mainly distributed in the HGF-PI3K/Akt-NF*κ*B signaling pathway (HGF, MET, PKB/Akt, p21, NF-*κ*B, and COX-2), the EGFR/FGFR/IGFR-MAPK signaling pathway (EGFR, FGFR, IGFR, PLCy, PKC, Ras, MEK, and ERK) and other related downstream signal transduction pathways. In addition to apoptosis, the final impact of these targets in a cancer cell is mainly associated with angiogenesis, proliferation, and genomic damage. Importantly, most of these targets or pathways, such as Bax [24], NF-*κ*B [25], MAPK pathway [26], EGFR [27], IGFR [28], and COX-2 [29], have been confirmed to be modulated by EGCG. However, as shown in Figure 3, these targets can be modulated by not only EGCG but also other polyphenols, which is in agreement with the “multicomponent, network targets” model.

### 3.6. Antidiabetic Mechanism

**Figure 6:**
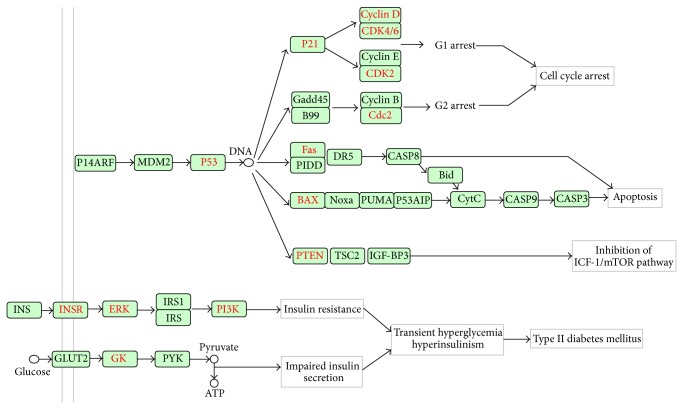
Simplified pathways in diabetes. All of the targets are shown by the gene name. The GTP-regulated targets are labeled in red.

Various studies have shown the beneficial effects of green on diabetes [30]. Japanese researchers have showed that drinking more cups of green tea can reduce the risk of diabetes by 33% [31]. Moreover, individuals who have habitually consumed tea for a long time present lower body fat, which is the property that is most related to diabetes [32]. Although several mechanisms have been proposed to explain the positive effect of green tea on diabetes [30, 33], these have not been confirmed. Fortunately, we identified three pathways (p53 signaling pathway, neurotrophin signaling pathway, and type II diabetes mellitus pathway) related to diabetes from all 147 pathways. Among these, the p53 signaling pathway and the neurotrophin signaling pathway were also found in the list of enriched pathways with *P* values of 2.39 × 10^−4^ and 1.68 × 10^−3^, respectively. Even though the *P* value found for the type II diabetes mellitus pathway was less than the set significance level, it still was selected for further analysis because it is closely correlated with diabetes. We constructed a diabetes-related pathway (Figure 6) by integrating these three pathways. As shown in Figure 6, 22 targets (colored in red) could be modulated by the GTPs. Previous studies on the p53 pathway have proven that hyperglycemia with diabetes promotes myocyte apoptosis mediated by the activation of p53 [34], which can be modulated by compound** 6**. Downstream of p53, GTPs can affect the cell cycle by mediating the targets p21, cyclin D, CDK4/6, and Cdc2. Moreover, the apoptosis of cells can be modulated by GTPs through targeting Fas and BAX. The ICF-1/mTOR pathway driving insulin resistance and diabetic complications [35] can also be mediated by GTPs through targeting its upstream protein PTEN. In the type II diabetes mellitus pathway, the proteins INSR, ERK, and PI3K contributing to insulin resistance are affected by the GTPs. In addition, the key protein GK (HK1), which is associated with the conversion from glucose to ATP and thereby contributes to impaired insulin secretion through the induction of mitochondrial dysfunction, can be regulated by compound** 3**.

### 3.7. Antineurodegenerative Disease Mechanism

**Figure 7:**
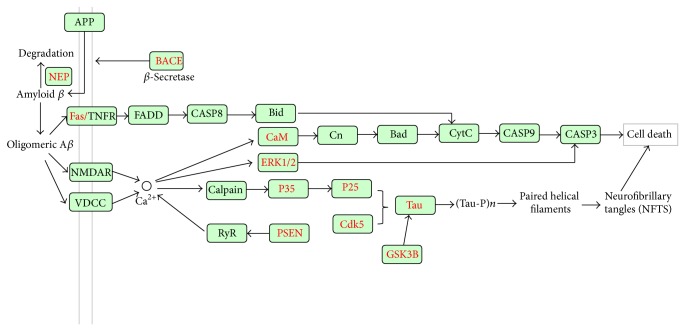
Simplified pathways in neurodegenerative disease. All of the targets are shown by the gene name, and the GTP-regulated targets are labeled in red.

Neurodegeneration, which occurs in Parkinson's, Alzheimer's, and other neurodegenerative diseases, appears to be multifactorial, in which a complex set of toxic reactions, including oxidative stress (OS), inflammation, reduced expression of trophic factors, and accumulation of protein aggregates, lead to the demise of neurons. One of the prominent pathological features is the abnormal accumulation of iron on top of the dying neurons and in the surrounding microglia [36]. Tea flavonoids (catechins) have been reported to penetrate the brain barrier and to protect against neuronal death in a wide array of cellular and animal models of neurological diseases [36, 37]. In this analysis, we identified two enriched pathway, namely, the Fc epsilon RI signaling pathway and Fc gamma R-mediated phagocytosis, related with brain iron accumulation. Fifteen proteins in these pathways can be regulated by the GTPs (Table S2). Although the *P*value found for Alzheimer's disease pathway was less than the set significance level, some of its proteins can be regulated by the GTPs. Alzheimer's disease (AD) is a progressive neurodegenerative disorder that is pathologically characterized by the deposition of *β*-amyloid (A*β*) peptides as senile plaques in the brain. EGCG has been proven to reduce A*β* generation [38]. Moreover, many key targets in Alzheimer's disease pathway can also be mediated by the GTPs. As shown in Figure 7, the *β*-site APP cleaving enzyme 1 (BACE), which promotes the formation of A*β* by cleaving APP, can be regulated by the GTPs. The membrane metalloendopeptidase (NEP), which inactivates the degradation of A*β*, can also be targeted by the GTPs. Evidence from several studies has indicated that the hyperphosphorylation of the Tau protein is responsible for its loss of biological activity and its resistance to proteolytic degradation and likely plays a key role in neurofibrillary degeneration in AD [39, 40]. Based on our analysis, the Tau proteins and their upstream proteins (p35, p25, PSEN, Cdk5, and GSK3B) can be modulated by GTPs. In addition, three proteins (Fas, CaM, and ERK1/2) leading to cell death can be modulated by GTPs.

### 3.8. Anticardiovascular Disease Mechanism

Previous epidemiological, clinical, and experimental studies have established a positive correlation between green tea consumption and cardiovascular health. Catechins have been proven to exert vascular protective effects through multiple mechanisms, including antioxidative, antihypertensive, anti-inflammatory, antiproliferative, antithrombogenic, and lipid lowering effects [41]. Therefore, green tea plays a role in anticardiovascular diseases by affecting many pathways shared with cancer, diabetes, and inflammation. In this study, only one pathway, namely, the vascular smooth muscle contraction pathway, was found to be directly related to cardiovascular diseases. Eight proteins (Cyt p450, PLA, CaM, MLCK, PKC, MEK, PKA, and MLCK) can be targeted by the GTPs, which can thus affect myosin (Figure S2).

### 3.9. Antimuscular Disease Mechanism

Duchenne muscular dystrophy (DMD) is a progressive muscle wasting disease that leads to early disability and death [42]. The disease is characterized by the absence of the dystrophin protein from the inner surface of the muscle cell sarcolemma. Muscle cells lacking dystrophin undergo cycles of degeneration and regeneration and are considered susceptible to contraction-induced injury [43]. In particular, the satellite cell proliferative capacity is exhausted, and the muscle fibers are replaced by connective and adipose tissue, resulting in a progressive loss of force generating capability [44]. Previous research studies have suggested that green tea can improve muscle health by reducing or delaying necrosis through an antioxidant mechanism [45]. In addition to this mechanism, we identified one pathway, the chemokine signaling pathway, related to macular degeneration. Through this pathway, GTPs can mediate the apoptosis, migration, survival, and growth, and differentiation of macular cells by affecting the PI3K/Akt/NF-*κ*B signaling pathway (Figure S3). Moreover, the key target Cdc42, which regulates the actin cytoskeleton, can be affected by compounds** 4** and** 5**, which may promote the differentiation and migration of muscle cells.

### 3.10. Anti-Inflammation Mechanism

The anti-inflammation activity of green tea has been demonstrated in many studies. Previous research has mainly focused on EGCG, which can disturb many inflammation-related pathways, such as the MAPKs, AP-1, and NF*κ*B pathways and STAT signaling [46]. In addition to these pathways, we identified an additional pathway, namelym the B cell receptor signaling pathway, related to inflammation and immunity. In this pathway, nine proteins (BTK, SYK, Ras, MEK1/2, Erk, PI3K, AKT, GSK3*β*, and NF*κ*B) that contribute to the immune response can be modulated by GTPs (Figure S4).

## 4. Conclusions

Green tea is commonly associated with traditional beverage rituals and particular lifestyles and is currently considered a source of dietary constituents endowed with biological and pharmacological activities that result in potential benefits to human health. Indeed, the novel pharmacological activities of green tea are arousing interest in the possible clinical use of green tea components for the prevention and treatment of several diseases. Although numerous studies are attempting to elucidate the molecular mechanisms through which green tea exerts its diverse biological activities, particularly its anticancer activity, a systematic understanding of the mechanisms through which green tea reduces disease risk is necessary to establish its efficacy for the population for which it could be most useful. The favorable properties of green tea have been ascribed to the high content of polyphenolic flavonoids. It is possible that different GTPs act on different targets in the signaling network of complex disease, resulting in a synergistic effect, which is in accordance with the holistic philosophy of network pharmacology that follows the “multicomponent, network target” model. Therefore, to systematically and comprehensively understand the mechanisms responsible for the diverse biological activities of green tea, a network pharmacology approach was used to analyze the “drug-target-pathway-disease” interaction network, which are constructed by collecting all of the confirmed and potential targets of GTPs. Six classes of diseases experienced beneficial therapeutic effects by green tea, as was determined at the molecular and signaling pathway levels based on the interaction network, which provides a better understanding of how the multiple ingredients in green tea act in synergy and the effects that these can have on multiple targets of a disease.

Moreover, this research study provides comprehensive and useful data for more in-depth studies of mechanisms of action of green tea. In addition, the understanding of the cell signaling pathways and the molecular events leading to a therapeutic effect will provide further insight for the identification and development of potent agents that specifically target these pathways.

## Supplementary Material

Figure S1: Node degree distribution of the protein interaction network induced by GTPs.
Figure S2-S4: GTPs mediated pathways in cardiovascular diseases, muscular disease and Inflammation, respectively.
Table S1: 200 Homo targets and interactions of “GTPs-Targets-Disease”.
Table S2: All pathways identified by pathway enrichment analysis and related targets/genes.

## Figures and Tables

**Table 1:** Enriched KEGG pathways.

Pathway ID	Term	Number of proteins	*P* value
hsa05214	Glioma	21	8.86*E* − 11
hsa05200	Pathways in cancer	39	2.41*E* − 09
hsa05215	Prostate cancer	22	8.26*E* − 09
hsa05223	Non-small-cell lung cancer	17	4.49*E* − 08
hsa05218	Melanoma	18	6.21*E* − 07
hsa04370	VEGF signaling pathway	15	9.00*E* − 06
hsa05212	Pancreatic cancer	13	5.89*E* − 05
hsa05213	Endometrial cancer	12	1.90*E* − 04
hsa04115	p53 signaling pathway	12	2.39*E* − 04
hsa05219	Bladder cancer	11	3.74*E* − 04
hsa04914	Progesterone-mediated oocyte maturation	13	8.84*E* − 04
hsa05222	Small cell lung cancer	14	8.84*E* − 04
hsa04664	Fc epsilon RI signaling pathway	14	8.84*E* − 04
hsa04510	Focal adhesion	20	1.16*E* − 03
hsa04722	Neurotrophin signaling pathway	18	1.68*E* − 03
hsa05220	Chronic myeloid leukemia	13	2.45*E* − 03
hsa04660	T cell receptor signaling pathway	15	3.03*E* − 03
hsa04012	ErbB signaling pathway	14	4.66*E* − 03
hsa04666	Fc gamma R-mediated phagocytosis	11	6.31*E* − 03
hsa04520	Adherens junction	9	7.74*E* − 03
hsa04662	B cell receptor signaling pathway	11	1.28*E* − 02
hsa05221	Acute myeloid leukemia	10	1.58*E* − 02
hsa00140	Steroid hormone biosynthesis	8	2.15*E* − 02
hsa04912	GnRH signaling pathway	15	2.43*E* − 02
hsa05210	Colorectal cancer	9	2.99*E* − 02
hsa05211	Renal cell carcinoma	11	3.47*E* − 02

**Table 2:** Diseases affected by GTPs and related pathways.

Disease	Related pathway
Cancer	Glioma (hsa05214)^a^
	Pathways in cancer (hsa05200)^a^
	Prostate cancer (hsa05215)^a^
	Non-small-cell lung cancer (hsa05223)^a^
	Melanoma (hsa05218)^a^
	Pancreatic cancer (hsa05212)^a^
	Endometrial cancer (hsa05213)^a^
	Bladder cancer (hsa05219)^a^
	Small cell lung cancer (hsa05222)^a^
	Acute myeloid leukemia (hsa05221)^a^
	Colorectal cancer (hsa05210)^a^
	Renal cell carcinoma (hsa05211)^a^

Diabetic	p53 signaling pathway (hsa04115)^a^
	Type II diabetes mellitus (hsa04930)^b^

Neurodegenerative disease	Fc epsilon RI signaling pathway (hsa04664)^a^
	Fc gamma R-mediated phagocytosis (hsa04666)^a^
	Alzheimer's disease (hsa05010)^b^

Cardiovascular diseases	Vascular smooth muscle contraction (hsa04270)^b^

Muscular disease	Chemokine signaling pathway (hsa04062)^b^

Inflammation	B cell receptor signaling pathway (hsa04662)^a^

^a^ *P* value < 0.05; ^b^ *P* value > 0.05.
