# Supplementary material for: Systematic Analysis of the Multiple Bioactivities of Green Tea through a Network Pharmacology Approach
Source: Evid Based Complement Alternat Med. 2014 Nov 30;2014:512081. doi: 10.1155/2014/512081 (PMC4267163; doi:10.1155/2014/512081)

**Supporting Information**

**Systematically analyze the mechanisms of green tea’s pleiotropic bioactivities by network pharmacology**

Shoude Zhang^1^, Lei Shan^2^, Qiao Li^1^, Xia Wang^1^, Shiliang Li^1^, Yuan Zhang^1^, Jianjun Fu^1^, Xiaofeng Liu^1^, Honglin Li^1,*^, Weidong Zhang^1,2,*^.

1. Shanghai Key Laboratory of New Drug Design, State Key Laboratory of Bioreactor Engineering, School of Pharmacy, East China University of Science and Technology, Shanghai 200237, China.

2. School of Pharmacy, Second Military Medical University, Shanghai 200433, China.

**Corresponding Authors**

E-mail: [wdzhangy@hotmail.com](mailto:wdzhangy@hotmail.com) (W. D.); [hlli@ecust.edu.cn](mailto:hlli@ecust.edu.cn) (H. L.); [shanleicn@126.com](mailto:shanleicn@126.com) (L. S). Phone/Fax: +86-21-81871244 (W. D.).

**Contents**

**Figure S1**. Node degree distribution of the protein interaction network induced by GTPs. The power law formula is: Y=25.869x-0.746, R-squared=0.705.

**Figure S2.** GTPs mediated pathways in cardiovascular diseases. This pathway graph was drawn according to vascular smooth muscle contraction pathway (hsa04270). Only the signal pathways regulated by GTPs were showed. All targets showed as gene name. Targets regulated by GTPs were labeled with red.

**Figure S3.** GTPs mediated pathways in muscular disease. This pathway graph was drawn according to chemokine signaling pathway (hsa04062). Only the signal pathways regulated by GTPs were showed. All targets showed as gene name. Targets regulated by GTPs were labeled with red.

**Figure S4.** GTPs mediated pathways in Inflammation. This pathway graph was drawn according to B cell receptor signaling pathway (hsa04662). Only the signal pathways regulated by GTPs were showed. All targets showed as gene name. Targets regulated by GTPs were labeled with red.

**Table S1**. 200 Homo targets and interactions of “GTPs−Targets−Disease”.

**Table S2**. All pathways identified by pathway enrichment analysis and related targets/genes.

Figure S1


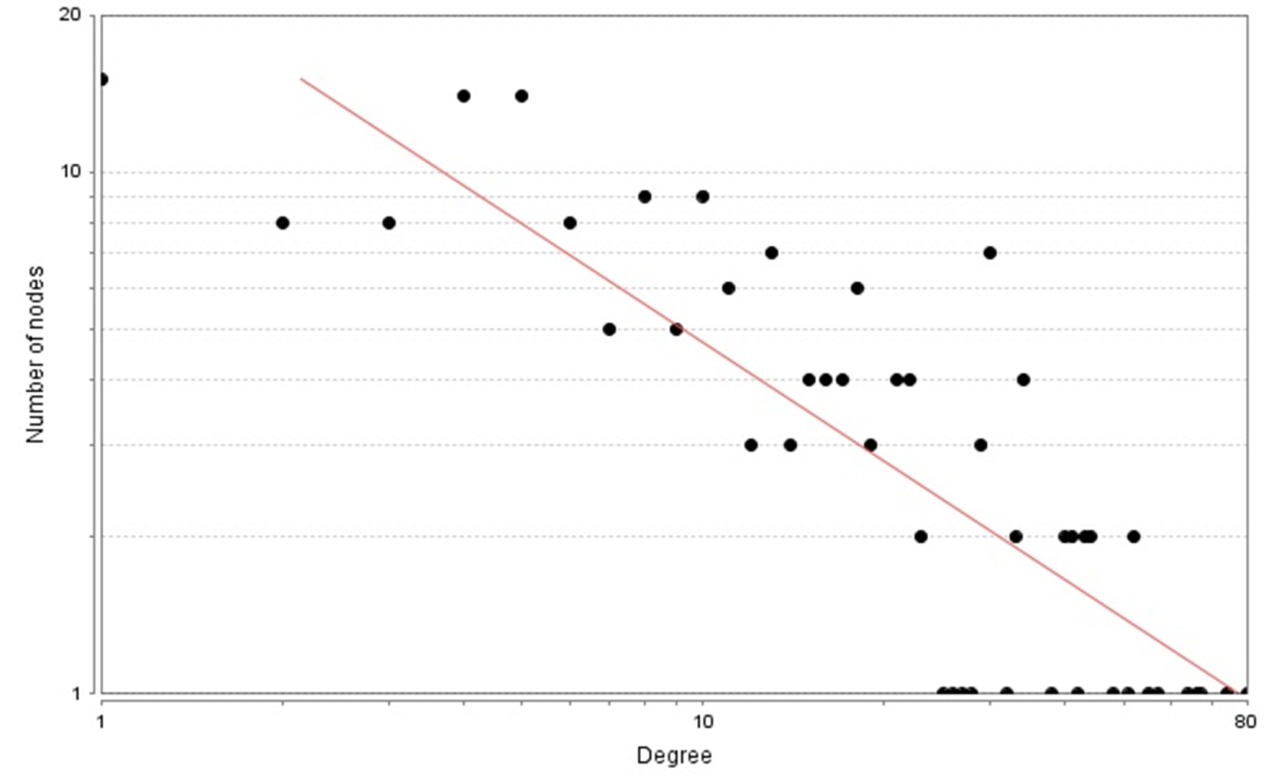


Figure S2


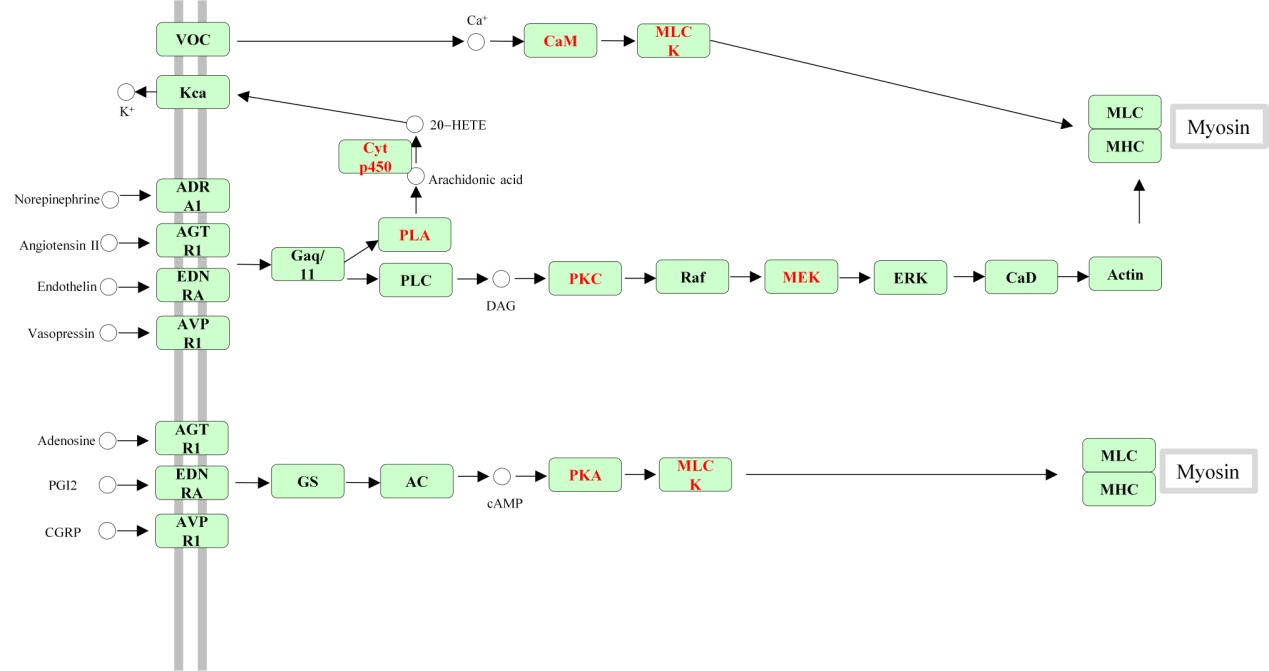


Figure S3


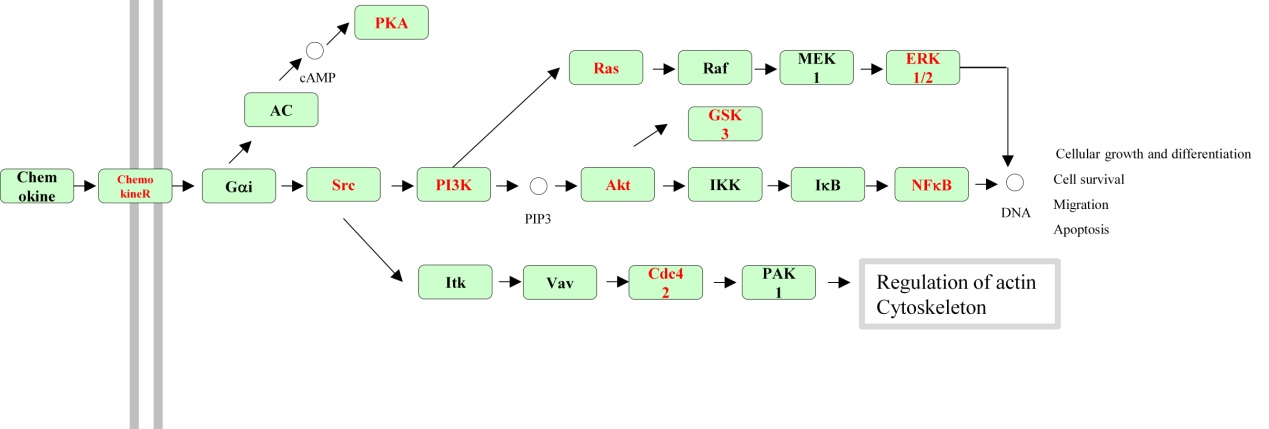


Figure S4


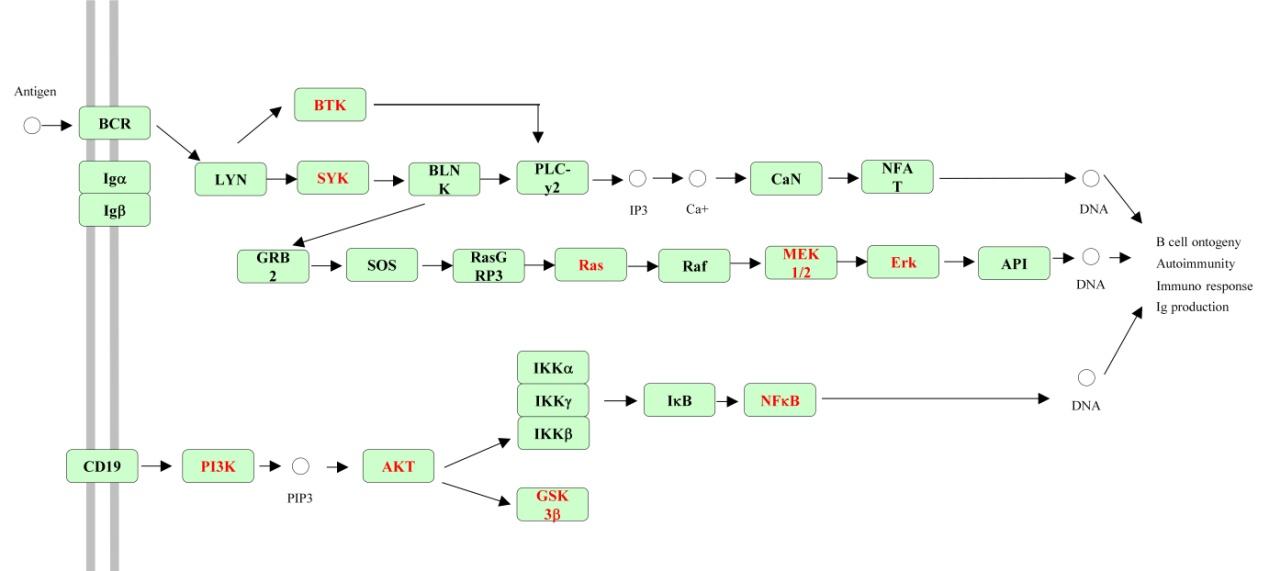

Supplement: Supplementary file 1 — Figure S1: Node degree distribution of the protein interaction network induced by GTPs. Figure S2-S4: GTPs mediated pathways in cardiovascular diseases, muscular disease and Inflammation, respectively. Table S1: 200 Homo targets and interactions of “GTPs-Targets-Disease”. Table S2: All pathways identified by pathway enrichment analysis and related targets/genes. [file 512081.f1.zip › SI.docx]
